# An Unusual Case of Resistant Hypokalaemia in a Patient with Large Bowel Obstruction Secondary to Neuroendocrine Carcinoma of the Prostate

**DOI:** 10.1155/2017/2394365

**Published:** 2017-03-13

**Authors:** Umasankar Mathuram Thiyagarajan, A. Ponnuswamy, A. Bagul, A. Gupta

**Affiliations:** ^1^Department of Hepatobiliary Surgery, Royal Free Hospital, Pond Street, London NW3 2QG, UK; ^2^Department of Paediatrics, Eastbourne District General Hospital, Eastbourne BN21 2UD, UK; ^3^Department of Transplantation, Leicester General Hospital, Leicester LE5 4PW, UK; ^4^Department of General Surgery, St Helier Hospital, Carshalton SM5 1AA, UK

## Abstract

Neuroendocrine Carcinoma of the Prostate (NECP) is rare and only few cases have been reported, constituting less than 0.5% of prostatic malignancies. We report a rare case of large bowel obstruction from NECP posing a further challenge in management due to resistant hypokalaemia. A 70-year-old man presented with clinical signs of large bowel obstruction who was known to have prostatic carcinoma three years ago, treated initially with hormone therapy then chemoradiation. The blood profile showed a severe hypokalaemia and CT scan revealed liver and lung metastases apart from confirming large bowel obstruction from local invasion of NECP. Severe hypokalaemia was believed to be caused by paraneoplastic syndrome from tumor burden or by recent administration of Etoposide. Intensive potassium correction through a central venous access in maximal doses of 150 mmol/24 hours under cardiac monitoring finally raised serum potassium to 3.8 mmol/L. This safe period allowed us to perform a trephine colostomy at the left iliac fossa. The postoperative period was relatively uneventful. This first case report is presenting a rare cause of large bowel obstruction from a neuroendocrine carcinoma of prostate and highlights the importance of an early, intensive correction of electrolytes in patients with large tumor burden from NECP.

## 1. Introduction

Neuroendocrine Carcinoma of the Prostate (NEC) is rare and only few cases have been reported constituting less than 0.5% of prostatic malignancies [1]. Epstein et al. classified the NEC into (i) usual prostatic carcinoma with NE differentiation; (ii) prostatic carcinoma with Paneth cell NE differentiation; (iii) carcinoid tumor; (iv) small cell carcinoma (SmCC); (v) large cell neuroendocrine carcinoma (LCNEC); and (vi) mixed NE carcinoma (SmCC or LCNEC), acinar adenocarcinoma [2].

Rectal involvement in prostatic only occurs in about 4% of patients [3–6]. The rectal infiltration can present as an anterior rectal mass (52%), an annular stricture (45%), and separate metastasis (3%) [7]. To the best of our knowledge this is a first case of LCNEC with rectal involvement; more importantly it presented with resistant hypokalaemia in our patient which posed a challenge before trephine colostomy. This electrolyte abnormality may have been caused by a paraneoplastic syndrome of NEC due to a large tumor volume and might have also contributed by the use of Etoposide based chemotherapy.

Hence, we feel that it is a unique and rare presentation of prostatic malignancy in a surgical patient; thus this report will be useful on managing challenges in patients with large bowel obstruction caused by NEC.

## 2. Case Presentation

**Figure 1:**
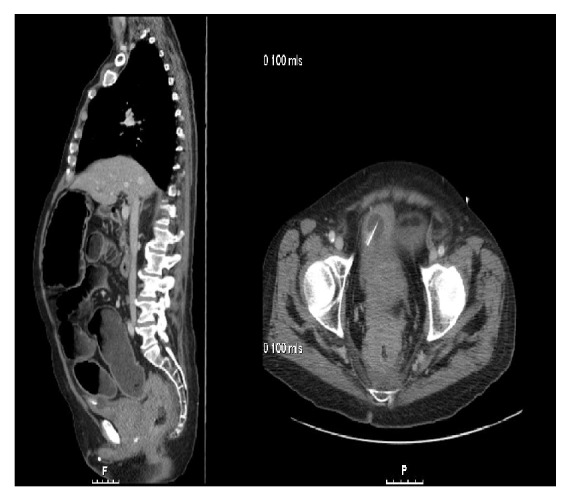
A sagittal and coronal view showing an advanced prostatic cancer involving rectum.

A 70-year-old man presented with abdominal distension, gurgling, and poor appetite for past three weeks. He has also experienced intermittent abdominal discomfort and nausea with reduction in stool volume. There was no history of vomiting but his stools were watery recently. He was diagnosed to have prostatic carcinoma three years ago, treated initially with androgen ablation for 1 year followed by chemoradiation as a part of clinical trial. Full details were not available as his prostate cancer treatment was performed in a tertiary oncology centre in another hospital.  Figure 1 shows an advanced prostatic cancer involving rectum.

He is hypertensive but suffered no other systemic illnesses. Due to the bladder outlet obstruction, he had had suprapubic catheter insertion two years back. He was retired 5 years back and lives with his wife.

## 3. Investigations

Preliminary investigations including full blood count (FBC), renal function tests (RFT), liver function (LFT), and clotting profile were performed. His blood tests showed a severe hypokalaemia (of 2.5 mmol/L) and remaining tests were within normal limits. Urine dipstick test was also normal.

Abdominal X-rays showed a dilated small, large bowel loops; a further abdominal computed tomography (CT scan) confirmed a large bowel obstruction possibly from local invasion of NEC. The transition point was noted in the pelvis just proximal to the soft tissue pelvis disease which encompassed the rectum resulting in significant narrowing. There were multiple liver and lung metastases with a single lytic lesion in the left femur noted.

## 4. Treatment

Patient was cannulated and intravenous morphine was given with cyclizine; intravenous saline (0.9%) was also commenced immediately for rehydration. As patient was suffering from hypokalaemia, intravenous potassium replacement is given with saline infusion through the peripheral line. In line with the CT findings, it was deemed that a trial conservative management with electrolytes correction would be the best choice. He was later reviewed by the palliative oncology team and has agreed with our plan.

Patient was kept nil by mouth and nasogastric tube; urinary catheter was inserted. The patient received trial phosphate enema but responded minimally. Later, patient was assessed by the anaesthetist team in the next day and central line was placed for rigorous intravenous potassium correction.

Daily FBC, RFT, and liver function tests were performed; but patient's hypokalaemia was failed to respond to a maximal dose (10 mmol/hour) ward based parenteral potassium infusion after 48 hrs. Patient symptoms also did not improve with conservative management. More importantly, he was pyrexial (39 degrees centigrade) and Tazocin® [(Piperacillin and Tazobactam) Pfizer Limited, Kent, United Kingdom] was started after consultation with the microbiologist. Due to the worsening of clinical picture, trial conservative management was abandoned and planned for emergency trephine colostomy.

Unfortunately patient's hypokalaemia (2.5 to 1.9 mmol/L) has got worse with new onset of hypomagnesaemia (0.55 mmol/L and hypophosphatemia (0.37 mmol/L)). As the ward based electrolyte correction did not improve, patient was transferred to surgical high dependency unit for intensive electrolyte correction with continuous cardiac monitoring.

Within 12 hrs, the potassium, other electrolytes have improved and we have taken this opportunity to perform a trephine colostomy. The ACTH level was measured postoperatively which was found to be elevated 100 pg/mL but unfortunately patient did not wish to proceed with any further investigation. Hence we cannot explain the source of high ACTH level with dexamethasone suppression test. Postoperative period was uneventful and patient was discharged in day 4 to attend his son's wedding. Now, it is three months after surgery, doing well with no problems with colostomy.

## 5. Discussion

The least common manifestation of neuroendocrine (NE) differentiation in prostate cancer is the development of NE tumors, with either pure or admixed conventional adenocarcinoma [1]. NEC is a distinct clinicopathologic entity that typically manifests after long-term hormonal therapy for prostatic adenocarcinoma [1].

Neuroendocrine (NE) cells represent a third type of epithelial cell in benign prostatic glands and are a distinctly minor component after basal cells and secretory luminal cells. They are considered to be terminally differentiated, postmitotic cells that arise from a putative stem cell with a basal cell phenotype [8]. They lack androgen receptor expression and are thought to play a regulatory role in proliferative and secretory activity of prostatic glandular epithelium [9, 10].

Large cell neuroendocrine carcinoma (LCNEC) of prostate subtype is exceptionally rare [11]. It is a high-grade tumor that consists of large nests and ribbons of cells with abundant pale to amphiphilic cytoplasm, large nuclei, and prominent nucleoli along with high mitotic activity and foci of necrosis [12, 13]. LCNEC is strongly positive for CD56, CD57, Chromogranin A, synaptophysin, and P504S/alpha methylacyl CoA racemase. Unfortunately as previous investigations and treatment for his prostate cancer were performed in another tertiary oncology unit, we could not get histological images for this publication.

Patients with NEC of prostate may present with haematuria, burning nocturia, urinary frequency, oliguria, or symptoms of urinary retention [1]. On the other hand, patients with “carcinoid-like” tumors are usually asymptomatic, although some cases of Cushing's syndrome induced by the production of adrenocorticotropic hormone have been reported [14].

LCNEC were most commonly an incidental finding at the time of palliative transurethral resection of prostate (TURP) for urinary obstruction in the setting of androgen-independent disease [1]. These tumors manifested as androgen-resistant disease and pursued an aggressive clinical course characterized by widespread metastases, a generally poor response to platinum-based chemotherapy, and a mean survival of 7 months after the presence of LCNEC was recognized [1].

Recent report on LCNEC showed a short survival which is likely reflecting the fact that the LCNEC was incidentally detected late in the course of the disease [1]. These patients nonetheless had a generally poor response to standard NEC chemotherapy protocols [1] as in our patient.

Here, our patient developed the large bowel obstruction long after the diagnosis of LCNEC. As we are aware of the metastatic peritoneal, liver, lung spread we have attempted a conservative management. Unfortunately this plan has failed with worsening of hypokalaemia which was a challenge before the surgical management.

Initial ward based intensive hypokalaemia correction attempt (up to 150 mmol/L per day) has failed. But, interestingly hypokalaemia has worsened with new onset of hypomagnesaemia and hypophosphatemia. Paraneoplastic Cushing's syndrome in which adrenocorticotropic hormone (ACTH), or corticotrophin-releasing factor, is produced by nonendocrine tumors is mostly associated with SCC of the lung [15]. It is not surprising that the syndrome has also been described in SCC of the prostate [16]. Small cell variant of NEC is known to cause electrolyte abnormalities, but LNEC has never been reported to have any similar presentations [17]. The above electrolytes abnormalities and high ACTH level are likely caused by LNEC but recent use of Etoposide might have also contributed for worsening of hypokalaemia. As patient did not wish to be investigated further due to the advanced prostatic malignancy, we could not confirm the aetiology of his resistant hypokalaemia.

The main lesson here was that electrolytes correction in these difficult patients has to be planned early and needs an intensive correction in the high dependency unit with continuous cardiac monitoring. Failure to recognize these potential challenges ahead could significantly increase the morbidity and mortality.

We believe that our case report will make clinicians, surgeons, urologists, anaesthetist, and intensivist aware of rarity and our experience could be translated into treating similar patients with large bowel obstruction.
